# Low genetic and phenotypic divergence in a contact zone between freshwater and marine sticklebacks: gene flow constrains adaptation

**DOI:** 10.1186/s12862-017-0982-3

**Published:** 2017-06-06

**Authors:** Susanne Holst Pedersen, Anne-Laure Ferchaud, Mia S. Bertelsen, Dorte Bekkevold, Michael M. Hansen

**Affiliations:** 1 Department of Bioscience, Aarhus University, Ny Munkegade 114, DK-8000 Aarhus C, Denmark; 2 National Institute of Aquatic Resources, Technical University of Denmark, Vejlsøvej 39, 8600 Silkeborg, Denmark; 3 Present address: Département de Biologie, Institut de Biologie Intégrative et des Systèmes (IBIS), Pavillon Charles-Eugène-Marchand, Université Laval, Québec City, QC Canada

**Keywords:** Adaptive divergence, Gene flow, Spatial autocorrelation, Sex-biased dispersal, Single nucleotide polymorphisms, Body shape, Eda, Hybrid zone

## Abstract

**Background:**

Distinct hybrid zones and phenotypic and genomic divergence is often observed between marine and freshwater threespine sticklebacks (*Gasterosteus aculeatus*). Nevertheless, cases also exist where marine-freshwater divergence is diffuse despite seemingly similar environmental settings. In order to assess what characterizes these highly different outcomes, we focused on the latter kind of system in the Odder River, Denmark. Here, a previous study based on RAD (Restriction site Associated DNA) sequencing found non-significant genome-wide differentiation between marine and freshwater sticklebacks. In the present study, we analyzed samples on a finer geographical scale. We assessed if the system should be regarded as panmictic, or if fine-scale genetic structure and local selection was present but dominated by strong migration. We also asked if specific population components, that is the two sexes and different lateral plate morphs, contributed disproportionally more to dispersal.

**Results:**

We assessed variation at 96 SNPs and the Eda gene that affects lateral plate number, conducted molecular sex identification, and analyzed morphological traits. Genetic differentiation estimated by F_ST_ was non-significant throughout the system. Nevertheless, spatial autocorrelation analysis suggested fine scale genetic structure with a genetic patch size of 770 m. There was no evidence for sex-biased dispersal, but full-plated individuals showed higher dispersal than low- and partial-plated individuals. The system was dominated by full-plated morphs characteristic of marine sticklebacks, but in the upstream part of the river body shape and frequency of low-plated morphs changed in the direction expected for freshwater sticklebacks. Five markers including Eda were under possible diversifying selection. However, only subtle clinal patterns were observed for traits and markers.

**Conclusions:**

We suggest that gene flow from marine sticklebacks overwhelms adaptation to freshwater conditions, but the short genetic patch size means that the effect of gene flow on the most upstream region must be indirect and occurs over generations. The occurrence of both weak unimodal and strong bimodal hybrid zones within the same species is striking. We suggest environmental and demographic factors that could determine these outcomes, but also highlight the possibility that long-term population history and the presence or absence of genomic incompatibilities could be a contributing factor.

**Electronic supplementary material:**

The online version of this article (doi:10.1186/s12862-017-0982-3) contains supplementary material, which is available to authorized users.

## Background

Selection is a key force enabling the adaptation of populations to local environments [1, 2]. Nevertheless, selection must be seen in conjunction with gene flow and drift, and it is well established that gene flow may constrain local adaptation [3–6]. When selection is combined with gene flow across populations, this may lead to the establishment of clines. It has been suggested that disruptive/divergent selection acting along an environmental gradient can gradually sharpen clinal variation until a single ancestral population ultimately fragments into two or more species [7]. Conversely, clines may occur when previously isolated populations come into secondary contact and establish hybrid zones [8, 9]. The shape and width of clines are strongly affected by gene flow or more specifically dispersal, defined as the geographical distance between the sites of reproduction by parents and their offspring [8]. Low dispersal rates and strong disruptive selection will essentially generate narrow clines whereas high dispersal and/or weak selection will generate wide clines. Additionally, a distinction has been made between bimodal hybrid zones, where parental genotypes predominate and only few hybrids are observed, and unimodal hybrid zones, where most genotypes represent hybrids [10]. It has been suggested that different types of selection may underlie these different categories, with prezygotic isolation being particularly important for bimodal zones [9].

The geographical scale over which dispersal occurs within a life-time is an important factor for understanding interactions between gene flow and selection. Furthermore, different groups of individuals within a population may contribute disproportionally to dispersal. Sex-biased dispersal is often observed in vertebrates, with male- or female biased dispersal resulting from the specific mating systems and/or extent of resource competition [11, 12]. However, phenotypic differences not ascribed to sex may also affect the propensity for dispersal of individuals, such as intraspecific differences in body shape in salmonid fishes adapted to short- or long-distance migration [13, 14]. In the context of clinal patterns, non-random dispersal exhibited by different morphs and ecotypes therefore could have important consequences.

The threespine stickleback (*Gasterosteus aculeatus*) is an excellent model for studying clines and hybrid zones, as it shows pronounced adaptation to different environments that can be identified both at phenotypic and genomic levels [15–17]. Strong divergence is often observed between marine and freshwater sticklebacks, and this type of system has received considerable attention as a model for understanding adaptive processes [17–23]. Marine sticklebacks are assumed to represent the ancestral form of the species, and in numerous instances freshwater populations have been founded by immigration of marine sticklebacks, which have adapted to freshwater conditions [15, 16, 24]. This is manifested in a number of phenotypic traits, such as marine sticklebacks being generally larger and exhibiting streamlined body shapes, whereas freshwater sticklebacks are smaller, deeper-bodied and with larger heads [25–28]. Some of these body shape differences may reflect phenotypic plasticity rather than genetically based adaptation [29, 30]. However, the most studied morphological difference between marine and freshwater sticklebacks concerns the number of lateral plates, which has a clear genetic basis [31]. Hence, marine sticklebacks typically show full armor (full-plated morphs), most freshwater populations show reduced numbers of lateral plates (low-plated morphs), and the gene Ectodyplasin-A (Eda) on chromosome 4 has been identified as a major quantitative trait locus (QTL) [32–34]. The suggested selective agents include fish predation in marine environments selecting for full-plated morphs, predation by invertebrates favoring low-plated morphs in freshwater, along with abiotic factors such as Calcium concentration [34].

In most cases when transitions between pairs of marine and freshwater populations have been studied, clear bimodal distributions of lateral plate numbers have been observed. Molecular markers have shown that this corresponds to narrow hybrid zones with bimodal distributions of genotypes and strong differentiation between parental populations [19, 22, 27, 35]. Important exceptions nevertheless exist, as full-plated morphs are in some cases widely distributed in freshwater environments [36, 37], and particularly in coastal regions [38–40]. Two recent studies of sticklebacks in the North Sea region of Belgium and the Netherlands suggest that this is an outcome of gene flow overwhelming selection [40, 41]. Hence, Raeymaekers et al. [40] investigated temporal dynamics of lateral plate and Eda variation in near-coastal freshwater populations and concluded that selection favored low-plated morphs and the associated Eda allele, but gene flow from marine sticklebacks nevertheless exceeded selection. Konijnendijk et al. [41] reached a similar conclusion based on analysis of gene-associated microsatellite markers that showed limited footprints of selection in this system despite being outliers in other marine-freshwater population pairs. Finally, a third recent study [42] analyzed marine and freshwater populations in Denmark using RAD sequencing [43]. In one system, the Odder River which flows into the Kattegat Sea, the population was dominated by full-plated morphs. Genome-wide differentiation was non-significant between marine sticklebacks and samples from the river, and genomic regions of elevated differentiation potentially under selection were essentially absent. Hence, this system resembles that studied by Raeymaekers et al. [40] and Konijnendijk et al. [41], and similar dynamics may be acting.

It is striking that both distinct bimodal hybrid zones and cases of weak, almost undetectable divergence have been observed within the same species and across comparable environmental transition zones. Detailed analysis of cases of weak genetic divergence despite strong environmental differences could therefore provide further insights into the factors that lead to different outcomes of hybrid zone dynamics. In the present study, we focused on sticklebacks in the Odder River system. We sampled sticklebacks from the marine environment and from the river, extending from the river mouth until ca. 8 km upstream. We analyzed morphological traits along with variation at 96 SNPs and an indel marker in close linkage with the Eda gene. In addition, we used a sex-specific genetic marker for determining the sex of individuals. We addressed the following research questions:

1. How are we to consider cases like the Odder River system? Is it a completely panmictic population with no local adaptive responses, or is fine-scale genetic structure and local selection present but dominated by strong migration? Based on detailed spatial sampling and spatial autocorrelation analysis [44] we analyzed genetic structure. We furthermore analyzed clinal variation at traits and markers and conducted outlier tests for genetic markers. These analyses allowed us to characterize the system and test the hypothesis that gene flow by marine sticklebacks overwhelms selection, even several kilometres upstream.
2. Do specific population components contribute more to dispersal in hybrid zones than others? In sticklebacks, full-plated morphs show reduced growth relative to low-plated morphs [45], and it is possible that this could also translate into differences in dispersal between morphs. Also, threespine sticklebacks are polygynous, which should favour male-biased dispersal [12]. On the other side, however, a trait such as the spiggin glue excreted and used by males for nest-building may be adapted to local water chemistry [46], which should pose an immediate selective cost for males dispersing across environmental gradients [47]. Previous studies of sex-biased dispersal in sticklebacks have yielded contrasting results [47, 48]. We made use of a powerful approach based on spatial autocorrelation analysis [49] for testing sex- and plate-biased dispersal. This allowed us to test if differences in dispersal among individuals within populations could be a contributing factor towards creating strong hybrid zones in some systems, whereas absence of selection in other systems could lead to strong gene flow and weak divergence. Based on the answers to these questions we considered possible scenarios that can explain why distinct molecular and phenotypic differences between freshwater and marine sticklebacks are evident in some systems but not in others, and from a general perspective why hybrid zones occur in some settings but are weak and absent in others within the same species.

## Methods

### Study localities and sampling

**Fig. 1:**
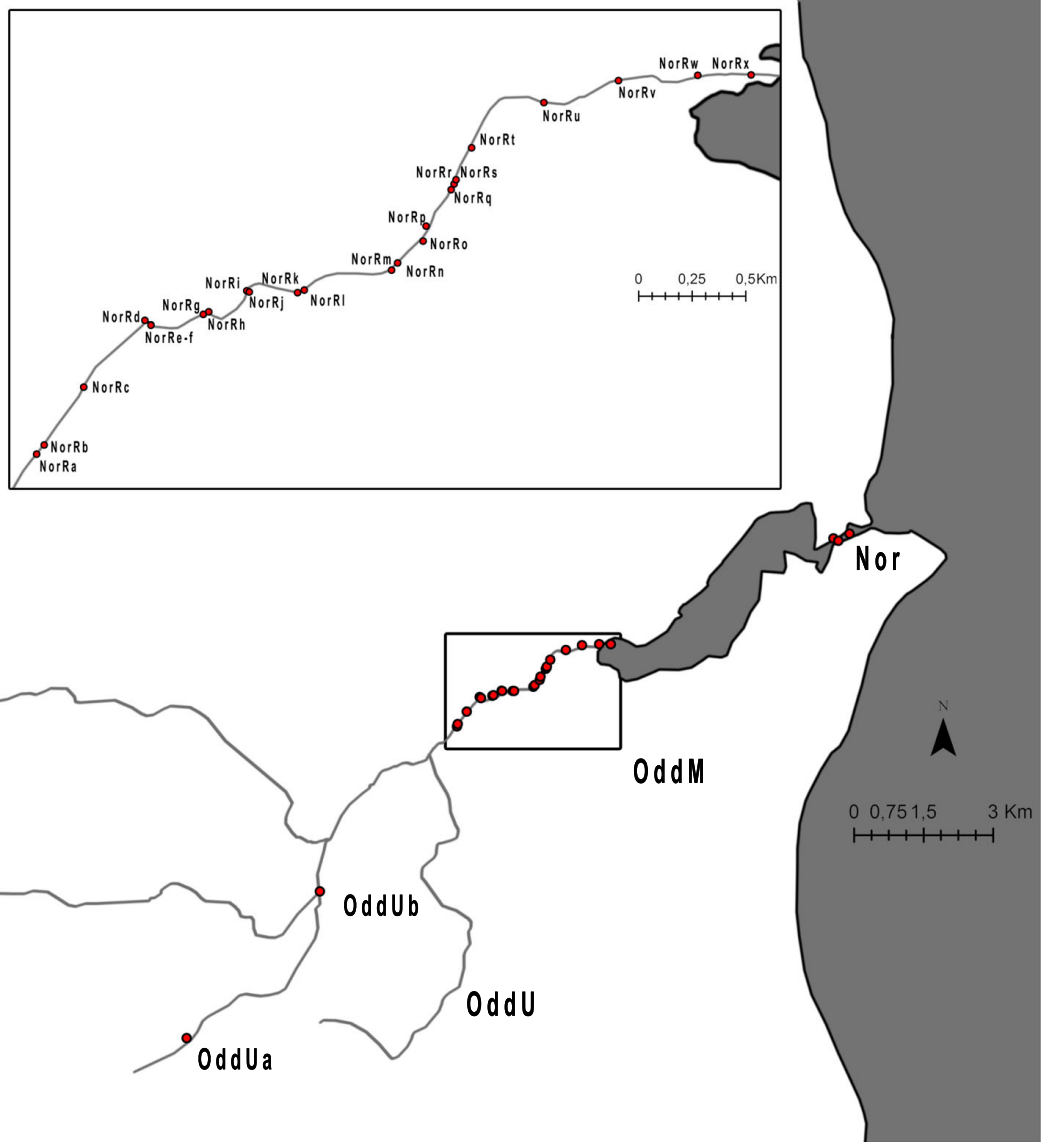
Sampling sites in the Odder River (freshwater) and Norsminde Fjord (marine). The three major groups of samples are as follows: (1) OddUa and OddUb are the two upstream localities in Odder River, pooled into the OddU group for some analyses. OddM denotes the group of samples that were collected continuously in the lower part of the river (as shown in the inserted box). Nor represents marine sticklebacks, sampled at three neighbouring localities and pooled into a single sample

The Odder River system is situated in Eastern Jutland, Denmark (Fig. 1). The river is ca. 16 km long and up to ca. 5 m wide and with no barriers to dispersal from the outlet to the most upstream sampled river stretches. Water depth in the downstream part is up to 1.5 m and in the upstream part up to 1 m. The lower ca. 500 m are influenced by tides, as evidenced by halting or even reversal of water currents during high tide. The fish fauna is dominated by threespine stickleback and brown trout (*Salmo trutta*). Intensive electrofishing conducted during this study also showed the presence of a few nine-spine sticklebacks (*Pungitius pungitius*) and single specimens of northern pike (*Esox lucius*) and European eel (*Anguilla anguilla*). The river flows into Norsminde Fjord, a shallow estuary with mean average depth of 0.60 m and about 3.5 km long and 0.5 km wide. It is characterized by shallow tidal flats, a deeper central channel and little vegetation. Salinity in the inner part coinciding with the river outlet ranges between 0.1‰ and 22.4‰, and between 5.2‰ and 26.2‰ in the outlet to the Kattegat Sea, depending on tides and occasional strong inflow from the sea caused by storms [50]. Visual inspection of the estuary during the spawning time suggested that it is not spawning habitat for threespine stickleback, but migration through the channel is clearly possible. The outlet of the fjord into the Kattegat Sea is regulated by a sluice. Threespine stickleback is highly abundant around the sluice and nesting males were observed in May–June.

A total of 390 threespine sticklebacks were sampled in the Norsminde Fjord (marine) and Odder River system (freshwater) from 2 May to 13 June 2013. This coincides with the spawning time of the species, increasing the probability that individuals were sampled near their sites of reproduction. Sampling was conducted with dip nets and cast nets (in the marine environment) and electrofishing and minnow traps (in freshwater). We defined the marine location, i.e. the outlet of the Norsminde Fjord into the Kattegat Sea as distance 0 m (Table 1). The outlet of the Odder River into Norsminde Fjord was at distance 3351 m. Fine-scale sampling was conducted from the outlet of the river and about 2.4 km upstream (distance 5756 m), with about one hundred meters separating each sampled locality (see Table 1 for details). In addition to the downstream region of the river, we sampled sticklebacks at two localities further upstream (distance 8702 and 11,627 m, respectively; see Table 1). The latter site represents the upstream limit of the distributional range of sticklebacks in the river. Electrofishing further upstream revealed no sticklebacks but a much denser population of brown trout than further downstream, and it is possible that the absence of sticklebacks is a result of intense predation by trout.

**Table 1:** Sampled localities, sample sizes (N), geographic coordinates and distance from the sea (Nor = 0)

Group Code	Locality Code	N	Longitude-latitude	Distance from the sea (in meters)
Nor	Nor	31	10.25974–56.02250	0
OddM	Norx	22	10.21657–56.01104	3351
	Norw	7	10.21433–56.01103	3493
	Norv	25	10.21102–56.01091	3702
	Noru	24	10.20788–56.01039	3915
	Nort	23	10.20485–56.00933	4182
	Nors	7	10.20420–56.00858	4276
	Norr	11	10.20412–56.00848	4288
	Norq	12	10.20400–56.00835	4305
	Norp	12	10.20295–56.00750	4420
	Noro	12	10.20282–56.00715	4459
	Norn	6	10.20184–56.00657	4549
	Norm	15	10.20158–56.00640	4573
	Norl	14	10.19785–56.00594	4828
	Nork	11	10.19755–56.00594	4838
	Norij	29	10.19543–56.00598	4984
	Norh	18	10.19383–56.00549	5124
	Norg	12	10.19360–56.00543	5136
	Noref	18	10.19141–56.00518	5288
	Nord	9	10.19116–56.00529	5308
	Norc	19	10.18866–56.00370	5542
	Norb	12	10.18693–56.00237	5736
	Nora	8	10.18673–56.00212	5756
OddU	OddUb	25	10.16012–55.98420	8702
	OddUa	8	10.13432–55.96830	11,627

The three major groups correspond to the pooling of the original samples for some aspects of statistical analysis: the marine (Nor), the downstream (OddM) and the upstream groups (OddU)

For most statistical treatments, we analyzed samples individually, but for some of the analyses we pooled samples into three major groups corresponding to the outlet of the fjord (Nor), the sections close to the river mouth (OddM), and the upstream part of the Odder River (OddU) (see Fig. 1 and Table 1 for details). Sampled sticklebacks were stored in 96% ethanol.

### Analysis of molecular markers

DNA was extracted from muscle tissue using the E.Z.N.A. DNA Tissue Extraction kit (OmegaBio-Tek, Norcross, GA, USA). Individuals were genotyped for 96 SNPs and an indel marker close to the Ectodysplacin (Eda) gene. The 96 SNPs represent a subset of 33,993 markers identified by RAD sequencing of a different set of Danish marine and freshwater threespine stickleback populations [51] and encompassed both markers under putatively directional selection in freshwater versus marine environments as well as putatively neutral markers. These SNPs do not necessarily coincide with genomic regions identified as marine-freshwater outliers in other studies [17, 20], and in general Danish stickleback populations do not to the same extent exhibit parallel evolution for the same genomic regions as observed in several other studies [42]. The genomic locations of the SNPs can be found in Ferchaud et al. [51]. The SNPs were genotyped on 96.96 Dynamic Arrays (Fluidigm Corporation, San Francisco, CA, USA), using the Fluidigm EP1 instrument according to the manufacturer’s recommendations. Variation at Eda was assessed using the Stn381 indel marker [33], and genotypes were determined by electrophoresis on a 3% agarose gel, followed by ethidium bromide staining and visualization under UV light. The sex of individuals was determined using primers targeting sex-specific variation in the Isocitrate dehydrogenase gene (IDH) [52]. Both sexes show a 302 bp band, but males show an additional 271 bp band. Bands were visualized as described for Stn381.

### Genetic structure and outlier tests

Observed (H_o_) and expected (H_e_) heterozygosity was calculated for the three major groups (Nor, OddM and OddU), and deviations from Hardy-Weinberg equilibrium were tested using exact tests [53]. Furthermore, we estimated genetic differentiation (F_ST_) between all three major groups and between pairs of groups using an unbiased estimator [54]. The significance of F_ST_ was estimated by permuting individuals between samples 10,000 times. ARLEQUIN 3.5.1.3 [55] was used for these analyses. In all cases of multiple tests, significance levels were adjusted using False Discovery Rate correction [56].

Genetic population structure was further assessed using STRUCTURE v.2.3.4 [57]. We assumed an admixture model, correlated allele frequencies, and we did not use any locality information. For estimating the most likely *k* we conducted runs assuming *k* = 1…7. Each run consisted of a burn-in of 10^5^ MCMC steps, followed by 2 × 10^5^ steps. Ten replicates were conducted for each *k*. We plotted the probability of the data [P(D)] using STRUCTURE HARVESTER [58].

We tested for possible selection acting on the molecular markers using an F_ST_-based outlier test implemented in the software LOSITAN [59]. We assumed a model of 10 populations, simulated 50,000 loci and used 95% confidence intervals and a false discovery rate of 0.1. The analysis was based on the three major groups of samples: Nor, OddM and OddU.

### Analysis of dispersal

We analyzed fine-scale structure using spatial autocorrelation analysis [60] implemented in GenAlEx 6.5 [44, 61]. This was based on a matrix of individual genetic distances [44] and a corresponding geographical distance matrix based on the waterway distance between samples (Table 1). We assumed geographical distance size classes of 100, 200 and 250 m and conducted the analysis based on the individuals sampled in the downstream part of the Odder River, OddM (Fig. 1), where sampling was continuous. The 95% confidence interval of distance-class specific *r* values and the 95% confidence interval in case of no spatial structure of individuals were estimated by bootstrapping 9999 times over pairs of individuals.

We furthermore assessed possible sex- and morph-biased dispersal. We divided the sampled individuals into two groups: a) males and females and b) full-plated and a combined group of low-plated and partially-plated individuals. We combined the latter two groups in order to obtain sufficient statistical power, as the majority of individuals were full-plated. We conducted spatial autocorrelation analysis on the same spatial scale as described above for each sex/morph separately, and then compared results for the two sexes and classes of morphs. Non-overlapping confidence intervals, particularly for the shortest distance classes would indicate sex- or morph-biased dispersal [49].

### Phenotypic analysis

For each individual, the lateral plates on the left side of the fish excluding keel plates (if present) as well as pelvic and dorsal spines, rays in the pectoral, dorsal, anal, and caudal fins were counted under a stereo microscope. Lateral plate morphs were defined as (I) low-plated: sticklebacks with 10 or fewer plates, (II) partially-plated: between 11 and 21 lateral plates, (III) fully-plated: > 21 plates [62]. As initial tests showed that the assumption of normal distribution of lateral plate counts was not met (with or without log-transformation data), a non-parametric Kruskal-Wallis test was conducted for testing differences in lateral plate counts between the three major population groups (Nor, OddM and OddU). All tests were conducted using JMP version 10.0.0 (SAS institute Inc., Cary, NC, 1989–2007).

**Fig. 2:**
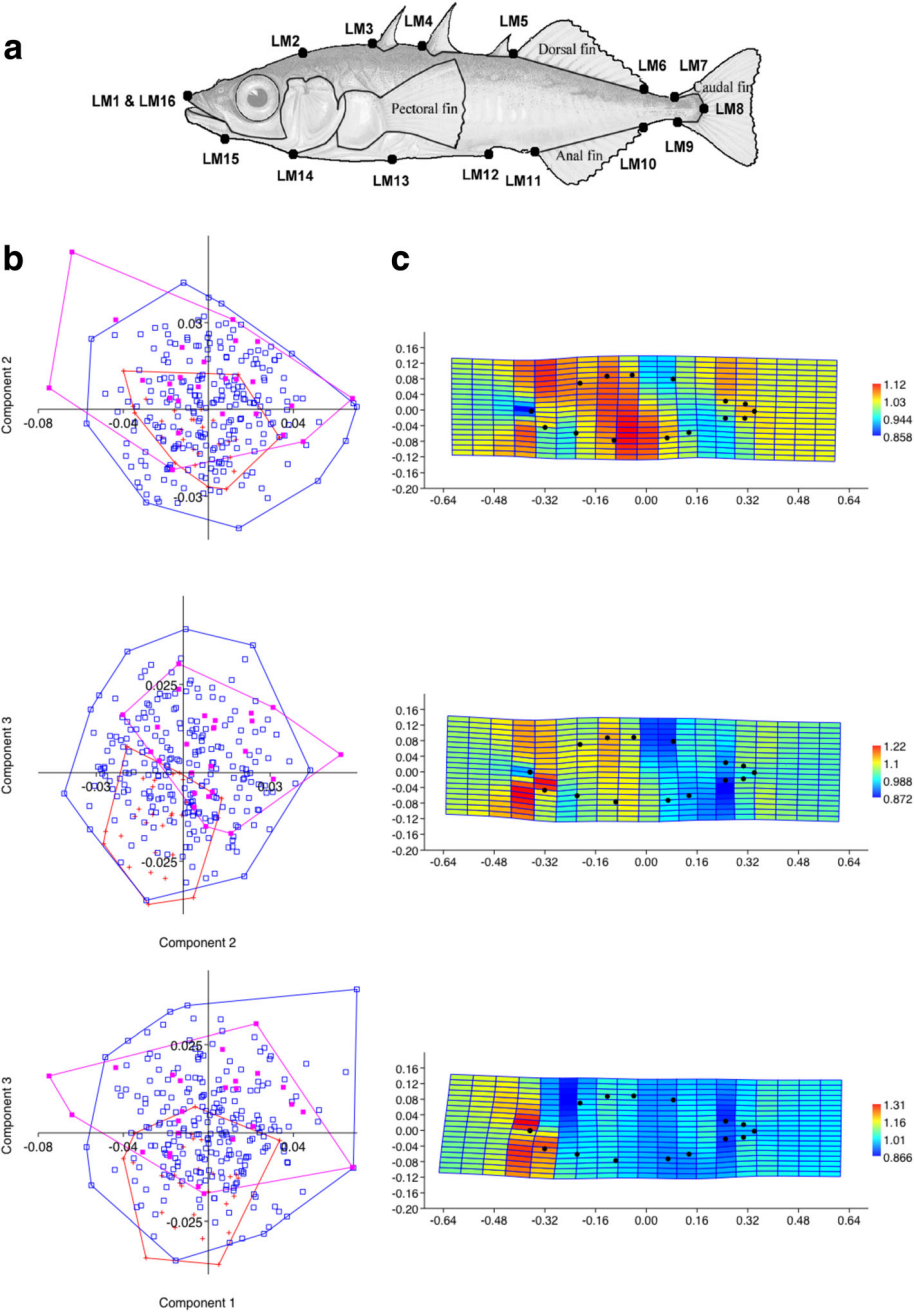
**a** The position of the 16 landmarks used. LM1: Anterior tip of the upper lip, LM2: Posterior extent of supraoccipital, LM3: Anterior insertion of first dorsal spine, LM4: Anterior insertion of second dorsal spine, LM5: Anterior insertion of dorsal fin on the dorsal midline (DML), LM6: Posterior insertion of dorsal fin on DML, LM7: Origin of the caudal fin membrane on DML, LM8: Posterior extent of caudal peduncle on lateral midline, LM9: Origin of the caudal fin membrane on ventral midline (VML), LM10: Posterior insertion of anal fin, LM11: Anterior insertion of anal fin, LM12: Caudal tip of the posterior process of the pelvic girdle on the VML, LM13: Posterior tip of the ectocoracoid, LM14: Anterior tip of the ectocoracoid, LM15: Posterior edge of the angular, LM16: Anterior tip of the upper lip (placed same place as LM1). **b** PCA scatter plot of the first and second, first and third, and second and third principal components that account for most of the variation in body shape. Marine individuals (Nor, *red*, plus sign), the downstream group (OddM, blue, empty squares) and the upstream group (OddU, *pink*, full squares) are shown in convex hulls. **c** Thin-plate spline transformation grid showing the body shape changes between the mean shapes of Nor to OddM (*top*), mean shapes of Nor to OddU (*middle*), and means shapes of OddM to OddU (*bottom*). The colored grids show the color-coded Jacobian expansion factors which measure the degree of local expansion or contraction of the grid: yellow to red for factors >1, indicating expansion; light to dark blue for factors between 0 and 1 indicating contraction

Geometric morphometric methods [63] were used to analyze body shape variation. A total of 311 specimens were photographed on their left side with a Canon 650D camera with a 60 mm lens, and two-dimensional coordinates were collected for 16 landmarks [64, 65] digitized on each specimen (Fig. 2a) using TpsDig2 version 2.17 [66]. The landmark data were aligned using the Procrustes superimposition method in PAST version 2.17c [67]. Variation in body shape was explored using principal component analysis (PCA). Jolliffe cut-off and broken stick methods were used to assess the number of principal components that should be considered significant [67]. Thin-plate spline transformation grids were used to visualize where the largest shape differences were found between the three populations. The mean shapes for each major group were first calculated in PAST version 2.17c. Subsequently, mean shapes of the major groups were plotted pairwise. The Jacobian color-code (yellow to red for factor > 1, indicating expansion; and light to dark blue for factors between 0 and 1 indicating contraction) were used to visualize changes in shape between localities. A one-way MANOVA (Multivariate ANalysis Of VAriance) was conducted to test for equality of means of the three populations (Nor, OddM and OddU) and a Canonical Variates Analysis (CVA) to test for separation between them. The centroid size, which corresponds to a Euclidian distance from all landmarks to the centroid, was extracted for each of the 311 individuals used for body shape analysis in PAST version 2.17c [67] and then tested across the three groups, using a Kruskal-Wallis test followed by a Steel-Dwass test.

### Analysis of parasite load

We focused on two general types of parasite infestation: a) parasites that live in the trunk of fishes, notably *Schistocephalus solidus* which is a widespread macro-helminth [68] and b) *black spot disease*, which is externally visible and can be caused by several trematode taxa: *Uvulifer, Crassiphilia, Cryptocotyle* and others [69]. The disease is seen as black spots on the skin that is caused by penetration of metacercariae followed by black pigmentation produced by the fish to surround the encysted metacercariae. Sticklebacks were classified as infected in the trunk if a parasite was visually observed or if the body cavity was grossly distended, which could resemble the distension caused by developing ovaries in females. However, in infected sticklebacks the body cavity bulges to the anterior as well as the posterior of the pelvis [69]. We did not attempt to identify the exact species underlying the two categories of parasites. The distribution of infection was tested across the three major population groups and pairs of these using a Pearson χ^2^ test, and Fisher’s exact test.

### Cline analysis at molecular and phenotypic levels

Using the Metropolis-Hastings Markov chain Monte Carlo algorithm implemented in the software HZAR [70] we applied likelihood functions for fitting molecular (allele frequencies) and morphological data to classic equilibrium models [71, 72]. In addition to molecular markers the following phenotypic traits were included: (1) the number of lateral plates, (2) the centroid size (as a measure of body size), (3–5) principal component values from axis 1, 2, and 3 explaining highest body shape variation, (6) infection of parasites in trunk, and (7) infection of *black spot disease*. We constructed two different data sets: (i) one including all individuals from the marine Nor location to the very upstream part of the river, OddU and (ii) another involving only the continuously sampled downstream part of the Odder river, OddM (319 individuals). The three cline models described in Brumfield et al. [73] were fitted to the molecular markers and the phenotypic traits as well as a null model. The null cline model assumes that allele frequency is independent of distance along clines. We ran three independent chains of 10^5^ iterations with a burn-in of 10^4^ and a separate seed for each model and then assessed convergence and stability by visualizing the MCMC traces. For each of the analyzed loci and phenotypic traits we plotted the maximum-likelihood clines for the best-fit model, selected according to the Akaike information criterion score [74].

## Results

### Molecular analysis

Analysis of SNPs and EDA indel marker variation provided results for all markers. However, significant drop-out of genotypes at markers 19,976 (71% missing data) and 26,062 (74% missing data) led to their omission. Sex was determined for 307 individuals; 31 individuals from Nor, 246 individuals from OddM and 30 individuals from OddU; due to depletion of DNA samples some individuals could not be analyzed. A total of 136 individuals were found to be males and 171 females.

### Genetic structure and outlier tests

Average expected heterozygosity (H_e_) across all loci was 0.211 in Nor, 0.216 in OddM and 0.255 in OddU, whereas observed heterozygosity (H_o_) was 0.203 in Nor, 0.212 in OddM and 0.221 in OddU. After False Discovery Rate correction and at a 5% significance level no loci showed deviations from Hardy-Weinberg equilibrium in Nor, 10 loci showed significant deviations in OddM (Eda, 5200, 15,358, 15,971, 31,101, 4073, 11,996, 30,997, 35,236 and 2620) and 6 showed significant deviations in OddU (5200, 13,744, 15,358, 21,688, 31,101 and 31,479). This represented 13 cases of deficits and 3 cases of excess of heterozygotes.

F_ST_ between the three major groups was −0.001 and non-significant (*p* = 0.935). Accordingly, pairwise F_ST_ were also low and non-significant: Nor-OddM F_ST_ = −0.003 (*p* = 0.999), Nor-OddU F_ST_ = 0.001 (*p* = 0.391), OddM-OddU F_ST_ = 0.001 (*p* = 0.302). STRUCTURE analysis of the entire data set showed the most likely number of distinct genetic clusters to be one (k = 1, see Additional file 1: Figure S1), and visual inspection of plots assuming k > 1 revealed no grouping of individuals (data not shown).

The LOSITAN outlier test encompassing all three geographical groups identified 5 outliers (at a 95% significance level) as candidates for being under directional selection: Eda, 7807, 7808, 27,791, and 33,942 (Additional file 1: Figure S2). Pairwise outlier tests identified six outliers between OddM and OddU (Eda, 7807, 7808, 14,236, 27,791, 33,942), three outliers between Nor and OddM (11,996, 14,236, 35,236) and seven outliers between Nor and OddU (Eda, 1800, 7807, 7808, 18,814, 27,791, 33,942). This suggests that differences between OddU furthest upstream in the Odder River versus the marine sample Nor and OddM close to the river mouth underlies the five outlier loci identified across all three major groups. In addition to the marker closely linked to Eda (Chromosome 4, position 12,800,313–12,810,434), the loci 7807 and 7808 are tightly linked and situated on Chromosome 8, positions 18,320,215 and 18,320,304, respectively. This does not correspond to a genomic region showing outlier status between marine and freshwater sticklebacks in previous studies [17, 20, 42]. The locus 27,791 is located on Chromosome 4, position 8,128,962. It corresponds to a region that shows overall high divergence between marine and freshwater populations in one study [17], but is not an outlier region in others [20, 42]. Finally, locus 33,942 is situated in Scaffold 309, position 4735 and has not been reported as an outlier region in previous studies.

### Analysis of dispersal

For the spatial autocorrelation analysis, we excluded 1) the markers under possible selection (Eda, 7807, 7808, 27,791, and 33,942), 2) three SNPs tightly linked to other SNPs (5812b, 30997b and 9202), 3) two SNPs with missing data in many individuals (19,976 and 26,062) and 4) five SNPs found on the sex chromosome 19 (15,358, 15,728, 15,971, 30,997 and 31,101). We found that a geographical distance size class of 200 m represented the best compromise between geographical resolution and numbers of individuals within classes.

**Fig. 3:**
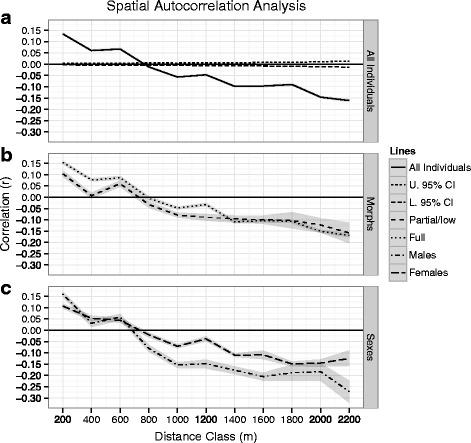
Corellograms showing the results of spatial autocorrelation analyses for the individuals sampled continuously from the river mouth and upstream. In all plots the shading along lines denotes 95% confidence intervals of r values. The analyses were conducted using GenAlEx 6.5 [61, 102]. **a** Analysis involving all individuals. The dashed lines denote the upper and lower confidence intervals for the case of no structure (i.e. correlation coeffificent *r* = 0; not shown in the other plots). **b** Separate corellograms for full-plated and low + partial-plated individuals. **c** Separate correlograms for male and females

For the analysis involving all individuals (Fig. 3a) the 95% confidence interval in case of no spatial structure of individuals was narrowly distributed around *r* = 0. This was also case for the analyses in Fig. 3b and c, but is not shown for clarity of presentation. We found a clear genetic structure involving all individuals (Fig. 3a); r values were positive and significantly higher than 0 for the shortest distance classes and then dropped below zero for the longer distance classes with an x-intercept at 770 m. This intercept can be interpreted as an estimate of the true extent of detectable positive spatial genetic structure or “genetic patch size” [60, 75]. Hence, the analysis revealed fine-scale genetic structure in sticklebacks with individuals showing non-random genetic relationships at a scale of up to ca. 770 m.

Figure 3b revealed higher r values for full-plated than for partial/low plated individuals, and 95% confidence intervals did not overlap for the shortest distance classes. The x-intercepts were 790 and 731 m for full and partial/low plated sticklebacks, respectively. Hence, full-plated individuals showed more dispersal although the differences were relatively small.

There was no evidence for sex-biased dispersal (Fig. 3c), as 95% confidence intervals of r values overlapped considerably for sexes in the shortest distance classes. The x-intercepts were 683 and 738 m for males and females, respectively, but with overlapping confidence intervals.

### Phenotypic analysis

Among a total of 390 sticklebacks only seven exhibited two instead of three dorsal spines, and three individuals exhibited four spines. Also, all except for a single individual exhibited two pelvic spines. There were no significant differences in the distribution of number of pelvic fin rays (Kruskal-Wallis test (K-W), H = 1.2092, df = 2, *p* = 0.5463), anal fin rays (K-W test, H = 3.8838, df = 2, *p* = 0.1431), dorsal fin rays (K-W test, H = 2.3146, df = 2, *p* = 0.3143) and caudal fin rays (K-W test, H = 1.2881, df = 2, *p* = 0.5252) between Nor, OddM and OddU (Additional file 1: Table S1).

Nor, OddM and OddU exhibited different distributions of numbers of lateral plates (Kruskal-Wallis test (K-W), H = 10.1642, df = 2, *p* = 0.0062*). A Steel-Dwass post hoc test showed significantly more lateral plates in Nor compared to OddU (Z = −2.74095, *p* = 0.0169) and OddM individuals (Z = −2.71738, *p* = 0.0181) (Additional file 1: Table S2). OddM and OddU were not significantly different (Z = − 1.53887, *p* = 0.2727), but OddU exhibited a trend for fewer plates (Additional file 1: Table S1 and Table S2). Full-plated morphs always had a keel (261/261), all but one of the partial-plated sticklebacks had a keel (95/96), whereas only 1/3 of the low-plated sticklebacks had a keel (11/33).

For the body shape analysis, the percentages of variance accounting for all the principal components are listed in Additional file 1: Table S3. According to the “scree plot” and “broken stick” methods, only the first three components were significant and therefore only these were considered further. The first three principal components account for 28% (PC1), 14% (PC2) and 11% (PC3) of the variation in body shape. The PCA scatter plots (Fig. 2b) indicated that the three geographical groups had different variances. Also, they overlapped to some extent. The marine group (Nor, red) exhibited lower variation than the two freshwater samples OddM and OddU. Further investigation of the loadings of each of these three components showed that on PC1 it was mainly the landmark LM8 that accounted for most variation, followed by LM12. On PC2 it was mainly LM11, and on PC3 mainly LM11 (see Additional file 1: Table S4 for loadings and Fig. 2a for definitions of the landmarks).

Thin-plate spline visualization indicated some body shape changes among pairs of the three major groups (Fig. 2c). The area around the head (LM 2, 3, 4) and pelvis (LM 12, 13), along with the area across the body depth tended to expand in OddM relatively to Nor (Fig. 2c, top). OddU contracted in shape mainly around the area of the tail (LM 6, 7, 8, 9), dorsal fin (LM 5, 6) and anal fin and tail (LM 10, 11) but expanded slightly in body depth when compared to Nor (Fig. 2c, middle). Finally, Fig. 2c (bottom) revealed a slight contraction in overall shape in OddU as compared to OddM. One-way MANOVA showed that multivariate differentiation among the three major groups was highly significant (Wilk’s lambda = 0.4237, df1 = 56, df2 = 520, F = 4.98, *p* = 1.108E-23). The results of the pairwise comparisons of the three groups showed that there was a significant difference in the means between all pairs (Nor vs. OddM, *p* = 7.36016E-15; OddM vs. OddU, *p* = 1.06573E-09; Nor vs. OddU, *p* = 0.0055459). The centroid size, which is a measure of body size, differed significantly across the three groups (Kruskal-Wallis, H = 40.1535, df = 2, *p* < .0001, see Additional file 1: Table S1). A non-parametric Steel-Dwass comparison of all pairs showed that Nor was significantly larger in centroid size than OddU and OddM (Z = −4.71840, *p* < .0001, Z = − 6.25459, *p* < .0001, respectively). No significant difference was detected between OddM and OddU (Z = −0.37508, *p* = 0.9254) (Additional file 1: Table S2).

### Parasite infestation

The frequency of infestation of parasites in the trunk was on average 23.3% (total *N* = 299) across all three major groups (Additional file 1: Table S5) and differences were non-significant (Pearson χ^2^ (2) =4.325, *p* = 0.1150, see Additional file 1: Table S5). The frequency of *black spot disease* was on average 58.46%, and significantly different across the three groups (Pearson χ^2^ (2) = 39.081, *p* < 0.0001) (Additional file 1: Table S6). Pairwise Fisher’s exact tests showed that this was due to significantly lower infection in OddU relative to both Nor and OddM (Additional file 1: Table S7).

### Cline analysis

For the cline analysis, we removed 4 loci (5812b, 30997b, 9202, 7807) that were closely physically linked to other of the loci. We also omitted 21 loci that presented an overall minor allele frequency < 0.05: 2114, 3231, 5238, 7745, 8466, 9206, 9990, 13,340, 14,236, 18,262, 20,063, 20,238, 21,688, 21,858, 22,037, 22,693, 23,341, 24,457, 30,542, 31,479, 33,387. Subsequently, the cline analysis was conducted independently for 69 loci. A cline was fitted for each of the 69 loci and 7 phenotypic traits, and the model with lowest AICc value was chosen (Additional file 1: Table S8).

**Fig. 4:**
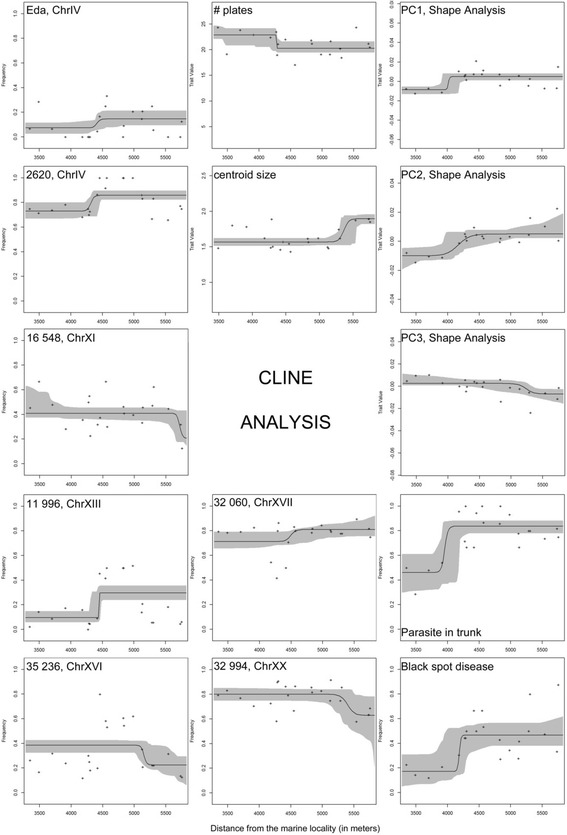
Cline analysis of the small scale-data set (from OddM only), showing results for the loci 2620, 11,996, 35,236, 16,548, 32,060, 32,994 and Eda and for the following quantitative traits: numbers of plates, centroid size, PC1, PC2, PC3 (of body shape variation), parasites in trunk and black spot disease. Plots show the maximum-likelihood cline and observed frequency data over the associated fuzzy cline region (95% credible cline region, indicated by *grey*). The distance indicates the distance to the sea (Nor = 0, see Table 1 for details). For each locality the mean was calculated and plotted

There were overall few loci that showed clinal patterns in either of the two data sets representing different geographical scales (all samples and the continuously sampled river stretch OddM, respectively). The following loci displayed clinal patterns at both geographical scales: 2620, 11,996 (which were also outliers between Nor and OddU), 35,236 (outlier between Nor and OddM), and 16,548 (Fig. 4); note that we only show the clines from the small geographical scale (OddM). In addition, at the small geographical scale (OddM) clinal patterns were observed for Eda, 32,060 and 32,994 (Fig. 4).

The following phenotypic traits displayed a cline at both geographical scales: number of lateral plates (decreasing number with increasing distance to the sea), centroid size, parasites (in trunk), and shape PC1 (principal components on 1. axis) (see Fig. 4). *Black spot disease* (parasite) showed a cline at the small geographical scale (OddM), with rate of infection increasing with distance from the sea but only a steep increase in the large data set rather than a specific clinal pattern (Fig. 4). PC2 and PC3 (body shape principal components) only displayed clines at the small geographical scale (OddM; see Fig. 4).

The clinal patterns suggested two possible cline centers: the first around 4100–4600 m from the sea (the loci EDA, 2620, 11,996, 32,060, parasite (trunk + *black spot disease*), plates, PC1, PC2, standard length), and the second around 5200–5400 m from the sea (the loci 16,548, 32,994, 35,236, centroid size, head length, PC3). Overall, the results indicate a shallow hybrid zone and/or change of selection regimes around 4100–5400 m from the sea in the lower part of the Odder River, corresponding to ca. 350–1500 m from the outlet of the river into the Norsminde Fjord.

## Discussion

The results of our study documented a system with very low and non-significant genetic differentiation between marine and freshwater sticklebacks sampled up to 8 km inland. Yet, despite low differentiation spatial autocorrelation analysis showed fine-scale genetic structure in the river with a genetic patch size of ca. 770 m. There was no evidence for sex-biased dispersal, but full-plated individuals showed higher dispersal compared to partial/low-plated individuals. At the phenotypic level, there was an increasing occurrence of shorter, deeper-bodied, larger-headed individuals and higher frequency of low-plated morphs in the upstream river (OddU) relative to downstream (OddM) and marine sticklebacks (Nor). Whereas trends for these traits are in the directions expected for differentiation between marine and freshwater threespine sticklebacks [19, 26, 33], it is nevertheless striking that even in the most upstream part of the river the prevalent plate morph was full-plated, and a concordant pattern was seen for the candidate locus Eda. There was some evidence for diversifying selection, particularly at the Eda locus. Also, some clinal patterns were evident at molecular markers and phenotypic traits, and there was concordance as to the geographical position of cline centres. Yet, all clines were shallow and for molecular markers they typically involved shifts of allele frequencies between 0.1 and 0.2 (Fig. 4).

In the following we consider how systems like Odder River sticklebacks can be characterized, we discuss the lack of sex-biased dispersal and the finding of plate morph-biased dispersal and its biological significance. Finally, we discuss possible reasons underlying the widely different patterns of marine-freshwater divergence observed in the stickleback system, which also parallels other findings of both bi- and unimodal hybrid zones within the same species [9].

### Characterization of system

We reject the hypothesis that the Odder River system and adjacent marine sticklebacks comprise a simple panmictic population. Even though analysis of tens of thousands of SNPs in a previous study [42] and analysis of hundreds of individuals using fewer SNPs in the present study yielded very low and non-significant F_ST_ values, the spatial autocorrelation analysis nevertheless showed fine-scale structure. Hence, with a genetic patch size of ca. 770 m the probability is low that individuals from e.g. the river mouth would interbreed with individuals 8 km upstream. This also leads us to rule out a simple explanation of the results showing overall low adaptive divergence throughout the system, that is anadromy. If all individuals migrated to sea and returned to freshwater to spawn, then they would all be subject to selection regimes characteristic of marine environments, which could explain the dominance of full-plated morphs. However, in order for the fine-scale structure to persist this would require accurate natal homing from the sea to specific sites in the river up to 8 km upstream. Transplantation and recapture of tagged sticklebacks in a small stream has demonstrated that they are able to home to the original point of capture when displaced up to 200 m downstream [76]. However, this involves ability to return to a site that the individual is familiar with and not specifically natal homing. The genetic patch size of 770 m corresponds roughly with findings of freshwater sticklebacks moving up to 1.8 km over one year [77]; the 770 m genetic patch size must be considered an average and does not exclude the possibility that some individuals could move further. This distance is much lower than the 8 km upstream where sticklebacks were found in the Odder River. Therefore, while it is likely that sticklebacks in the most downstream parts of the river are anadromous, this is unlikely to account for the whole population.

It is also evident that some diversifying selection is ongoing in the system. Although the possibility of false positives must be taken into account when interpreting outlier tests, it is reassuring that the well-known candidate locus Eda was the most significant outlier. Nevertheless, F_ST_ at this locus was relatively low (0.11); this corresponds to F_ST_ values of 0.12 and 0.10 observed in some previous studies [40, 78], but is considerably lower than differentiation observed in a number of other studies reviewed by Raeymaekers et al. [40], with F_ST_ ranging from 0.38 to 0.86. Even though clines at loci and traits were shallow, the fact that there was general concordance between the geographical positions of cline centers also suggests a response to an environmental gradient. Clines at some traits, notably infection by *black spot disease* could in principle reflect genetic adaption. However, they could also reflect differences in environmental conditions, such as salinity, which by themselves affect the abundance of the parasites without invoking adaptive responses. Geographical differences and clinal patterns for body shape variation may also reflect phenotypic plasticity [29, 30], but the differences in lateral plate numbers have a genetic basis [33].

In total, the system is best characterized as a diffuse, unimodal hybrid zone, where gene flow overwhelms selection. However, gene flow affects the most upstream localities indirectly, in a step-wise manner and over generations. This result illustrates the point previously made by Peakall et al. [75] that in continuous habitats (like the Odder River system), populations of widespread species may be connected via gene flow over considerably longer distances than the scale of dispersal within one or a few generations. Hence, a pronounced local genetic structure may be present, even if at the same time genetic differentiation measured as F_ST_ is low between geographically more remote sites. Although it may take some generations for marine-adapted alleles to travel to the most upstream part of the population, even low amounts of gene flow may constrain local adaptation. The classically inferred relationship between gene flow/migration (m) and selection (s) is that m > s will lead to gene flow overwhelming selection [79]. More elaborate models focusing on the constraints of gene flow on quantitative adaptive traits have been developed and show that even rates of m < 0.01 can have a constraining effect relative to the optimal phenotypic value [6, 80]. Whereas we did not attempt to estimate quantitative genetics parameters in our system, and the complexity of dispersal in the system and low differentiation does not allow for estimating gene flow, we nevertheless expect that the rate of gene flow from marine sticklebacks affecting the most upstream parts of the population is > > 0.01, potentially with a significantly constraining effect on local adaptation.

### Dispersal differences among population segments

Sex-biased dispersal might account for differences in introgression of mitochondrial DNA, sex chromosomes and autosomal loci observed across some hybrid zones, such as in salamanders (*Plethodon spp.*) [81] and field voles (*Microtus agrestis*) [82]. In threespine stickleback there are arguments supporting hypotheses of both male- and female-biased dispersal, and two different studies have provided evidence in both directions [47, 48]. Yet, we found no evidence for sex-biased dispersal, despite the fact that spatial autocorrelation was analyzed over an environmental gradient that could particularly incur fitness loss of dispersing males, if spiggin glue used for nest-building is adapted to different water chemistries [47]. Although our study represents just a single location, it is nevertheless interesting that no sex-biased dispersal was observed at a small geographical scale where dispersal clearly occurs and environmental heterogeneity exists. This raises the possibility that dispersal in threespine sticklebacks simply may not be sex-biased at all.

In contrast to sex-biased dispersal we did, however, observe different dispersal of lateral plate morphs. Spatial autocorrelation analysis suggested that full-plated morphs exhibited higher dispersal than partial and low-plated morphs. The differences were small, but on the other hand we pooled partial and low-plated morphs to obtain sufficiently high sample sizes, and it is possible that a direct comparison between only full- and low-plated sticklebacks would have revealed stronger differences. We find it surprising that full-plated morphs normally characterizing marine sticklebacks disperse more in freshwater, but it could reflect a continual influx of full-plated marine sticklebacks that is not counter-balanced by downstream migration of low-plated individuals. Also, a behavioral study of sticklebacks exhibiting different morphs and Eda genotypes showed that individuals exhibiting full-plated alleles showed preference for a range of salinities, mainly determined by the salinities they were already acclimated to [83]. Low-plated individuals, in contrast avoided intermediate salinities and preferred either fully marine or freshwater conditions. Whereas the precise biological significance of these differences could not be resolved, the findings are nevertheless congruent with our results showing higher dispersal of full-plated individuals along an environmental gradient. Differences in dispersal between plate morphs may therefore be a contributing factor towards the dominance of full-plated sticklebacks in the Odder River and other similar systems.

### Contrasting results across studies

Why do we see so distinct genetic and phenotypic differences between marine and freshwater sticklebacks in some [17, 19, 20, 22, 27, 35] but not in other studies [39–41], including the present? This question is reminiscent of the finding of both unimodal and bimodal hybrid zones within the same species, as in e.g. *Triturus* newt [84], *Corthicus* grasshopper [85] and *Bombina* toads [9, 86], indicating different roles of natural selection, pre- and postzygotic barriers in different settings. We consider four possible scenarios: 1) Extinction and recent recolonization of sticklebacks in the Odder River, 2) presence or absence of fish predators, 3) the migration-selection balance and differences in migration rate between systems, 4) allopatric divergence and secondary contact in some systems but not in others.

1. One obvious explanation of our results could be recent extinction of the freshwater population and recolonization by marine sticklebacks, and that adaptive divergence therefore is still in a process of establishment. However, we find it unlikely that a major extinction event should have occurred undetected. The Odder River has a large, indigenous self-sustaining population of brown trout. Although local extinction and recolonization in a few small tributaries has been recorded, the total population is stable and has never been stocked [87]. Hence, given the status of brown trout in the system there is no reason why sticklebacks should have experienced recent extinction-recolonization due to e.g. pollution events.
2. Another possible explanation concerns differences in fish predator composition across different freshwater environments. In the Odder River the dense brown trout population may pose a significant predator pressure on sticklebacks, which should favor full-plated phenotypes [34, 88]. European eel has also historically been an important fish predator in the system. Electrofishing in 1992 revealed the presence of numerous eels (Michael M. Hansen, personal observation) and the currently low number of eels likely reflects the general decline of the species [89]. In that sense selection regimes imposed by fish predators may be quite similar in the marine and freshwater environments of the study area. This would resemble conditions in the St. Lawrence River, Quebec, Canada, where fish predators are abundant [39]. In another related study, fish predators were observed in some, but not all of their study localities, and the authors did not emphasize this aspect [40].
3. Even if fish predation could explain the dominance of full-plated morphs and Eda alleles in the Odder River, it remains striking that low divergence between freshwater and marine sticklebacks was a genome-wide effect, as shown by RAD sequencing [42]. Fish predation represents an aspect of selection regimes that could be different or similar between freshwater and marine environments, but other biotic and particularly abiotic factors would for sure be different, such as salinity. Some physiological traits under differential selection could be complex and determined by many genes, the effects of which would be difficult to demonstrate using genome scans [90, 91]. However, the ATP1a1 gene on Chromosome 1 is strongly involved in osmoregulation and has been found to be a marine-freshwater outlier in several studies [17, 21, 23, 92, 93]. Yet, RAD data from showed that differentiation is virtually zero between Nor and OddU for this genomic region [42]. The overall very low genome-wide divergence points to gene flow from marine sticklebacks overwhelming selection, but it still remains unresolved why gene flow from marine sticklebacks can overwhelm selection in some cases but not in others. A possible and testable hypothesis could simply relate to the population sizes of marine populations adjacent to freshwater habitats; larger marine population sizes could lead to higher immigration rates into freshwater populations and a stronger constraining effect on selection as opposed to smaller marine populations causing lower immigration rates.
4. Divergence between different stickleback ecotypes has been argued to represent speciation with gene flow [94]. However, it has recently been suggested that most genomic islands of differentiation may instead reflect allopatric divergence [95], although this has not been specifically addressed in marine-freshwater sticklebacks. If this is indeed the case, then the presence of bimodal hybrid zones observed in some studies [19, 22, 35] could reflect secondary contact after allo- or parapatric divergence, possibly involving prezygotic barriers [96, 97], that has occurred e.g. in lakes or mediated by other types of environmental heterogeneity within the systems. The Odder River system does not encompass lakes, and stickleback habitat is continuous from the river mouth to the upstream distributional limit of the species, leaving little opportunity for allo- or parapatric divergence. However, the localities in the North Sea region and the lower St. Lawrence River in Quebec, Canada analyzed in other studies [39, 40] comprise larger and more heterogeneous areas with ample opportunities for divergence in allo/parapatry. Yet these studies found similar low divergence between marine and freshwater sticklebacks as in the present study, which does not lend support to this hypothesis.
  Nevertheless, an extended version of this scenario might be possible. It has been suggested that many instances of contact zones seemingly maintained by selection and adaptation to different environments may in fact represent endogenous genetic incompatibilities that interact with environmentally based selection [98]. The possibility of such incompatibilities increases when previously isolated groups or phylogeographic lineages make secondary contact. Coexistence and possible admixture of different phylogeographic lineages has been documented in e.g. the Pacific North-West, parts of Western Europe and Western Greenland [99, 100], thus providing potential for incompatibilities to be present. The Kattegat region, where the Odder River is situated appears to represent a single phylogeographical lineage [100]; it would be interesting to assess if regions with pronounced bimodal hybrid zones coincide with areas of pronounced secondary contact, and if regions with low marine-freshwater divergence are characterized by phylogeographical homogeneity.

## Conclusions

The Odder River represents a system where the freshwater stickleback population is only weakly divergent from marine sticklebacks at the phenotypic and genetic levels. It is nevertheless best characterized as a unimodal hybrid zone, where gene flow from marine sticklebacks strongly constrains adaptive divergence in the freshwater environment. The system exhibits a fine-scale genetic structure and genetic patch size that renders it unlikely that marine immigrants can affect the most upstream part of the river within a single generation. Instead, the effects of gene flow occur indirectly over several generations. Even though the presence of both uni- and bimodal hybrid zones has been documented in other species [9], freshwater and marine sticklebacks represent systems showing an almost extreme range of divergence, from weak unimodal to strong bimodal hybrid zones. Sticklebacks thus offer significant potential for understanding the factors that determine hybrid zone dynamics. In the present case, we identified unequal dispersal between different morphs as a factor that likely contributes to the dominance of full-plated morphs in the system. Among a range of factors that could lead to bimodal hybrid zones in some systems and unimodal in others, we suggest that the size of marine populations adjacent to freshwater habitats could determine immigration rates into freshwater, thereby possibly constraining effects on adaptation. We also advocate consideration of other factors than those strictly related to ecological and environmental factors, that is the possibility of secondary contact and endogenous incompatibilities in some systems and not in others [98].

## Additional file

Additional file 1: Table S1. Nonparametric Kruskal-Wallis tests for differences in morphological traits among samples of threespine sticklebacks. **Table S2.** Nonparametric comparisons for all pairs of samples (Nor, OddM, OddU) using the Steel-Dwass method. **Table S3.** The principal components for analysis of morphological traits (body shape landmarks), their eigenvalues, the percentage each account for, and 95% bootstrapped confidence (1000 bootstraps) for each. **Table S4.** Principal component loadings on the three first axes (PC1, PC2, PC3) for each of the 16 morphological landmarks (x and y coordinate). **Table S5.** Tests for differences in parasite infestation in the trunk among the major groups of samples. **Table S6.** Tests for differences in parasite infestation (*black spot disease*) in the trunk among the major groups of samples. **Table S7.** Fisher’s Exact Test for pairwise comparisons of the three major groups (OddM, Nor, OddU) and their level of parasite infection by *black spot disease*. **Table S8.** Cline analysis. Best fit models for SNPs and quantitative traits. **Figure S1.** Probability of the number of clusters (k) represented by the individual, estimated using STRUCTURE v.2.3.4. **Figure S2.** Results of outlier tests for the SNPs and Eda marker. (PDF 569 kb)
