# Supplementary material for: Low genetic and phenotypic divergence in a contact zone between freshwater and marine sticklebacks: gene flow constrains adaptation
Source: BMC Evol Biol. 2017 Jun 6;17:130. doi: 10.1186/s12862-017-0982-3 (PMC5461706; doi:10.1186/s12862-017-0982-3)

Additional File 1 for:

Low genetic and phenotypic divergence in a contact zone between freshwater and marine sticklebacks: gene flow constrains adaptation

Susanne Holst Pedersen[1], Anne-Laure Ferchaud[1,3], Mia S. Bertelsen[1], Dorte Bekkevold[2] and Michael M. Hansen[1*]

[1] Department of Bioscience, Aarhus University, Ny Munkegade 114, DK-8000 Aarhus C, Denmark

[2] National Institute of Aquatic Resources, Technical University of Denmark, Vejlsøvej 39, 8600 Silkeborg, Denmark

[3] Present address: Département de Biologie, Institut de Biologie Intégrative et des Systèmes (IBIS), Pavillon Charles-Eugène-Marchand, Université Laval, Québec City, QC, Canada

* Corresponding author, e-mail mmh@bios.au.dk

Supplementary information:

Table S1: Nonparametric Kruskal-Wallis tests. The table shows tests for differences of traits across the three major groups: Nor, OddM and OddU. The following traits were tested: number of lateral plates, and ray number in the pelvic, anal, dorsal, and caudal fin. The centroid size (as estimate for body size) was also tested. Score sum: records the sum of the rank score for each level. Expected score: records the expected score under the null hypothesis that there is no difference among class levels. Score mean: records the mean rank for each level. (Mean-Mean0)/Std0: records the standardized score. Mean0 is the mean score expected under the null hypothesis. Std0 is the standard deviation of the score sum expected under the null hypothesis. The null hypothesis is that the group means or medians are in the same location across groups. H is the chi-square test statistic.

Trait	Group	Count	Mean	SD	Score Sum	Expected Score	Score Mean	(Mean-Mean0)/Std0	H	DF	Prob> H
plates	Nor	31	23.6452	3.30200	7736.00	6060.50	249.548	2.814	10.1642	2	0.0062*
	OddM	326	21.3067	5.60146	63124.5	63733.0	193.633	-0.746			
	OddU	33	18.7576	7.32304	5384.50	6451.50	163.167	-1.741			
Pelvic fin	Nor	31	9.9677	0.179605	5839.00	6060.50	188.355	-1.068	1.2092	2	0.5463
	OddM	326	10.0092	0.214637	63987.5	63733.0	196.281	0.896			
	OddU	33	10.0000	0.000000	6418.50	6451.50	194.500	-0.153			
Anal fin	Nor	31	8.38710	0.715422	6870.00	6029.50	221.613	1.572	3.8883	2	0.1431
	OddM	324	8.18210	0.677493	61604.5	63018.0	190.137	-1.932			
	OddU	33	8.30303	0.683961	6991.50	6418.50	211.864	1.042			
Dorsal fin	Nor	31	11.0968	0.597486	6769.00	6029.50	218.355	1.384	2.3146	2	0.3143
	OddM	324	10.9136	0.766117	62002.0	63018.0	191.364	-1.389			
	OddU	33	11.0000	0.829156	6695.00	6418.50	202.879	0.502			
Caudal fin	Nor	31	11.9677	0.179605	5944.00	6045.00	191.742	-0.784	1.2881	2	0.5252
	OddM	325	11.9877	0.110425	63574.0	63375.0	195.612	1.131			
	OddU	33	11.9394	0.348155	6337.00	6435.00	192.030	-0.739			
Centroid size	Nor	31	8.19737	0.94362	7841.00	4836.00	252.935	6.324	40.1535	2	<.0001*
	OddM	254	6.63653	1.34951	37062.0	39624.0	145.913	-4.175			
	OddU	26	6.48977	1.44682	3613.00	4056.00	138.962	-1.008			

Table S2: Nonparametric comparisons for all pairs using the Steel-Dwass method. Several traits were compared between the three major groups (Nor, OddM, OddU). Score mean difference: gives the difference of the score means. Z: gives the standardized test statistic, which has an asymptotic standard Noral deviation under the null hypothesis. P-value: gives the asymptotic two-sided p-value for Z. Hodges-Lehmann: gives the Hodges-Lehmann estimator of location shift; the median of all paired differences between observations in the two samples. Upper and lower CL gives the confidence limit for the Hodges-Lehmann statistic.

Trait	Level	- Level	Score Mean Diff	Std Err Dif	Z	p-Value	Hodges-Lehmann	Lower CL	Upper CL
plates	OddU	Nor	-12,5748	4,58774	-2,74095	0,0169*	-2,00000	-8,00000	0
	OddU	OddM	-28,8655	18,75761	-1,53887	0,2727	-1,00000	-4,00000	0
	OddM	Nor	-52,0522	19,15531	-2,71738	0,0181*	-1,00000	-2,00000	0
Centroid size	OddU	OddM	-6.2538	16.67313	-0.37508	0.9254	-0.10868	-0.70652	0.559480
	OddU	Nor	-20.8269	4.41398	-4.71840	<.0001*	-1.49011	-2.25926	-0.867300
	OddM	Nor	-98.0705	15.67976	-6.25459	<.0001*	-1.47544	-2.04075	-0.965220

Table S3: The principal components, their eigenvalues, the percentage each account for, and 95% bootstrapped confidence (1000 bootstraps) for each. The % variance indicates how much of the body shape variation each PC accounts for. The first PCs will explain the highest variation.

PC	Eigenvalue	% variance	Eig 2.5%	Eig 97.5%
1	0.00053361	28.057	24.887	32.027
2	0.000266918	14.034	12.524	16.482
3	0.000209222	11.001	9.6597	12.647
4	0.000137071	7.2071	5.9113	8.7499
5	0.000123491	6.4931	4.873	8.2186
6	0.000108355	5.6972	4.4655	7.1451
7	9.90031E-05	5.2055	4.075	6.5186
8	7.75901E-05	4.0796	3.2488	4.8711
9	6.57826E-05	3.4588	2.7285	4.0353
10	5.55928E-05	2.923	2.3249	3.369
11	3.6333E-05	1.9104	1.4705	2.2564
12	3.20489E-05	1.6851	1.308	2.0446
13	2.85911E-05	1.5033	1.1641	1.7999
14	2.29862E-05	1.2086	0.934	1.4321
15	2.12325E-05	1.1164	0.88283	1.2961
16	1.65193E-05	0.86857	0.6708	0.96576
17	1.38169E-05	0.72648	0.54968	0.83397
18	1.22356E-05	0.64334	0.47603	0.72419
19	9.83639E-06	0.51719	0.39196	0.58222
20	6.71887E-06	0.35327	0.25642	0.39449
21	5.54775E-06	0.2917	0.21549	0.33841
22	5.36168E-06	0.28191	0.20191	0.32084
23	4.37502E-06	0.23003	0.16404	0.25058
24	4.08616E-06	0.21485	0.15205	0.24908
25	2.78915E-06	0.14665	0.10526	0.15679
26	1.72552E-06	0.090726	0.066923	0.095603
27	9.29923E-07	0.048895	0.032628	0.052721
28	8.5383E-08	0.0044894	0.0032928	0.004893
29	3.87568E-08	0.0020378	0.0014022	0.0022652
30	6.85422E-16	3.6039E-11	4.4987E-13	3.8071E-11
31	2.55307E-17	1.3424E-12	1.0058E-12	1.4403E-12
32	1.12765E-17	5.9291E-13	3.7804E-13	3.4502E-11

Table S4:

Principal component loadings on the three first axes (PC1, PC2, PC3) for each of the 16 landmarks (x and y coordinate).

	Axis 1	Axis 2	Axis 3
x1	-0.06468	-0.2857	-0.08432
y1	-0.2363	-0.009626	0.02227
x2	-0.1195	0.279	-0.01805
y2	0.04987	0.1633	0.01763
x3	-0.02386	0.3317	0.1766
y3	0.1823	0.1101	0.1231
x4	-0.02954	0.35	0.1557
y4	0.2534	-0.01087	0.1583
x5	-0.02524	0.01159	0.1711
y5	0.2982	-0.09056	0.2006
x6	0.02608	-0.0676	-0.03814
y6	0.0477	-0.00147	0.08326
x7	0.09755	-0.1128	-0.2048
y7	-0.2564	0.1067	-0.01328
x8	-0.0202	-0.02493	-0.2941
y8	-0.4357	0.1405	-0.0694
x9	-0.122	-0.06421	-0.3299
y9	-0.262	0.05653	0.006992
x10	-0.06394	-0.05303	-0.06842
y10	0.04127	-0.08885	0.05158
x11	-0.1102	-0.4597	0.5476
y11	0.257	-0.1911	0.006537
x12	0.1199	0.2261	0.3054
x12	0.3206	0.09098	-0.2982
x13	0.1952	0.2332	-0.05041
y13	0.1682	-0.02457	-0.2341
x14	0.1352	0.05157	-0.07172
y14	-0.0138	-0.1228	-0.05736
x15	0.06989	-0.1271	-0.1122
y15	-0.1769	-0.1198	-0.02163
x16	-0.06467	-0.2882	-0.08433
y16	-0.2375	-0.008407	0.02381

Table S5

Contingency of infection of trunk by major group. The result for this table was a non-significant relationship between group and infection level (Pearson χ^2 (2) = 4.325, p = 0.1150).

Count<br>Total %<br>Col %<br>Row %<br>Expected<br>Deviation<br>Cell Chi^2	**Not infected. 0**	**Infected. 1**	
**OddM**	28<br>7.18<br>9.36<br>90.32<br>23.7667<br>4.23333<br>0.7540	3<br>0.77<br>3.30<br>9.68<br>7.23333<br>-4.2333<br>2.4776	31<br>7.95
**Nor**	244<br>62.56<br>81.61<br>74.85<br>249.933<br>-5.9333<br>0.1409	82<br>21.03<br>90.11<br>25.15<br>76.0667<br>5.93333<br>0.4628	326<br>83.59
**OddU**	27<br>6.92<br>9.03<br>81.82<br>25.3<br>1.7<br>0.1142	6<br>1.54<br>6.59<br>18.18<br>7.7<br>-1.7<br>0.3753	33<br>8.46
	299<br>76.67	91<br>23.33	390

Table S6

Contingency table for infection of *black spot disease* by major group. The result indicated a significant relationship between the group and amount of infection (Pearson χ^2 (2) = 39.081, p<.0001*).

Count<br>Total %<br>Col %<br>Row %<br>Expected<br>Deviation<br>Cell Chi^2	**Not infected. 0**	**Infected. 1**	
**OddM**	7<br>1.79<br>4.32<br>22.58<br>12.8769<br>-5.8769<br>2.6822	24<br>6.15<br>10.53<br>77.42<br>18.1231<br>5.87692<br>1.9058	31<br>7.95
**Nor**	125<br>32.05<br>77.16<br>38.34<br>135.415<br>-10.415<br>0.8011	201<br>51.54<br>88.16<br>61.66<br>190.585<br>10.4154<br>0.5692	326<br>83.59
**OddU**	30<br>7.69<br>18.52<br>90.91<br>13.7077<br>16.2923<br>19.3643	3<br>0.77<br>1.32<br>9.09<br>19.2923<br>-16.292<br>13.7588	33<br>8.46
	162<br>41.54	228<br>58.46	390

Table S7

Fisher's Exact Test for pairwise comparisons of the three major groups (OddM, Nor, OddU) and their level of parasite infection by *black spot disease*. When parasites were present a value of 1 was given, when no infection a value of 0 was given. The null hypothesis is that no difference in proportions of parasite infection exists. The alternative hypothesis is given in the table.

	**Fisher's Exact Test**	**Prob**	**Alternative Hypothesis**
Nor vs. OddU, black spot disease	Left	<.0001*	Prob(black spot disease=1) is greater for group=Nor than OddU
	Right	1.0000	Prob(black spot disease=1) is greater for group=OddU than Nor
	2-Tail	<.0001*	Prob(black spot disease=1) is different across group
Nor vs. OddM, black spot disease	Left	0.0583	Prob(black spot disease=1) is greater for group=OddM than Nor
	Right	0.9767	Prob(black spot disease=1) is greater for group=Nor than OddM
	2-Tail	0.1180	Prob(black spot disease=1) is different across group
OddM vs. OddU, black spot disease	Left	<.0001*	Prob(black spot disease=1) is greater for group=OddM than OddU
	Right	1.0000	Prob(black spot disease=1) is greater for group=OddU than OddM
	2-Tail	<.0001*	Prob(black spot disease=1) is different across group

Table S8:

Cline analysis. Best fit models for SNPs and quantitative traits. Model with the lowest AICc value indicates the best model, and subsequently the selected model to plot the data along.

Locus	ln L (model selected)	AICc Model 1	AICc Model 2	AICc Model 3
L276	-9.363137	41.46580	26.77911*	35.25047
L1139	-11.47429	2392.068		2400.566
L1800	-13.68842	45.49375	35.42997*	43.62078
L1955	-14.83271	51.93834	37.71812*	46.35996
L2114	-11.63375	34.38635	31.32020*	39.45501
L2620	-39.77678	110.69910	87.60697*	96.06693
L3644	-16.03759	43.80067	40.12816*	48.12732
L3851	-7.34489	29.96388	22.74276*	31.17226
L4073	-7.849127	19.71411*	19.86839	28.01310
L4390	-13.66082	65.34744	35.37449*	43.51626
L4521	-6.489597	23.80267	21.03203*	29.19971
L5200	-13.716	38.35367	35.48569*	44.22701
L5812	-15.13347	34.28275*	35.73211	43.85190
L5939	-5.700661	22.95849	19.45430*	27.60860
L6844	-7.707532	26.21039	23.46805*	31.68220
L7275	-11.06665	33.42065	30.18643*	38.43841
L7808	-7.426464	26.47076	22.92107*	31.10536
L8351	-9.756588	30.59485	27.58132*	35.79778
L9026	-13.87464	39.18542	35.81743*	44.30092
L10131	-16.83566	53.73717	41.72416*	49.86158
L10561	-9.275505	31.17200	26.60442*	35.58952*
L11120	-9.760459	38.73145	27.57390*	35.27849
L11996	-60.91042	173.2275	129.8740*	139.4475
L13177	-14.86775	41.71349	37.78876*	45.95175
L13682	-9.991603	33.89683	28.03619*	36.20663
L13744	-10.04224	39.57084	28.13732*	36.27244
L14188	-8.782631	21.58107*	23.13475	32.04636
L15044	-11.06535	47.66596	30.18744*	38.34094
L15358	-20.87843	45.77267*	46.38702	54.13062
L15728	-12.55791	29.13167*	32.44644	39.99164
L15971	-10.05279	33.87510	28.15842*	37.54304
L16548	-15.69849	58.65110	39.45010*	47.33129
L16649	-10.76721	25.55028*	25.71377	33.84855
L16691	-7.694542	27.73083	23.44192*	31.49541
L17506	-15.62314	50.32274	39.29927*	47.28900
L17577	-9.1105	30.98516	26.27398*	34.41202
L17787	-11.67092	27.35768*	27.38190	35.51011

L18047	-11.52113	27.05803*	27.69528	35.42226
L18651	-6.137683	25.73596	20.32807*	28.46624
L18814	-15.35718	34.73016*	36.65656	44.75338
L20222	-7.837468	33.47168	23.72792*	31.87122
L20574	-9.662365	33.58065	27.37757*	36.15601
L20825	-9.17425	32.26912	26.40176*	34.53148
L21643	-9.82176	28.20644	27.69650*	35.57597
L21693	-14.1333	39.86128	36.31986*	44.44200
L22229	-11.55318	38.93212	31.15920*	39.28666
L22319	-11.45255	44.87197	30.95822*	39.13242
L23102	-11.89061	37.04057	31.83435*	40.14945
L25425	-12.1503	36.69627	32.35345*	40.49380
L26071	-7.063501	18.14277*	19.23059	27.35650
L26405	-12.08568	34.47066	32.22406*	40.19396
L27027	-9.515909	31.74587	27.08452*	35.22900
L27608	-15.90743	35.83381*	36.63048	45.36011
L27768	-8.208527	28.80429	24.47018*	32.61915
L27791	-18.5999	41.21673*	41.66885	49.78899
L28321	-12.24254	41.80611	32.53906*	40.66990
L28526	-11.71742	38.77249	31.48754*	39.55074
L28703	-8.887492	31.78899	25.82796*	33.97744
L29903	-10.05728	29.74793	28.17161*	36.67680
L30997	-10.98478	25.98549*	28.13890	36.55591
L31101	-21.06116	51.98317	50.17796*	58.32863
L31954	-11.4491	46.68974	30.95132*	39.09034
L32060	-16.85517	54.33170	41.76332*	50.38916
L32977	-7.992231	25.00876	24.03730*	32.02213
L32994	-11.71077	41.23058	31.47537*	40.04510
L33523	-10.74358	30.22601	29.54521*	37.92750
L33942	-13.83301	39.18401	35.71986*	43.45711
L35236	-47.25915	156.8297	102.5739*	111.6332
L35752	-11.79152	39.07562	31.63644*	39.92445
Eda	-38.84367	88.37293	85.74312*	93.08261

Quantitative trait	ln L (model selected)	AICc Model 1	AICc Model 2	AICc Model 3
plates	-1188.765	2391.829		2400.390
parasites spot	-17.74638	39.52353*	43.30574	50.09200
Parasites Trunk	-24.17636	85.48944	56.45661*	64.75749
Centroid	-81.59951	177.5687		186.0571
PC1	683.4467	-1336.123		-1343.944
PC2	726.1021	-1428.105		-1429.255
PC3	458.0237	-901.6502		-893.0341

Figure S1:

The Ln probability of there being K genetic clusters (±SD) in the entire data set of 390 individuals. Following the guidelines of Pritchard *et al.* (2000), the most likely value of K is interpreted to be the lowest value of K at the start of the plateau (in this case K=1). K=1 has the smallest SD and highest probability.

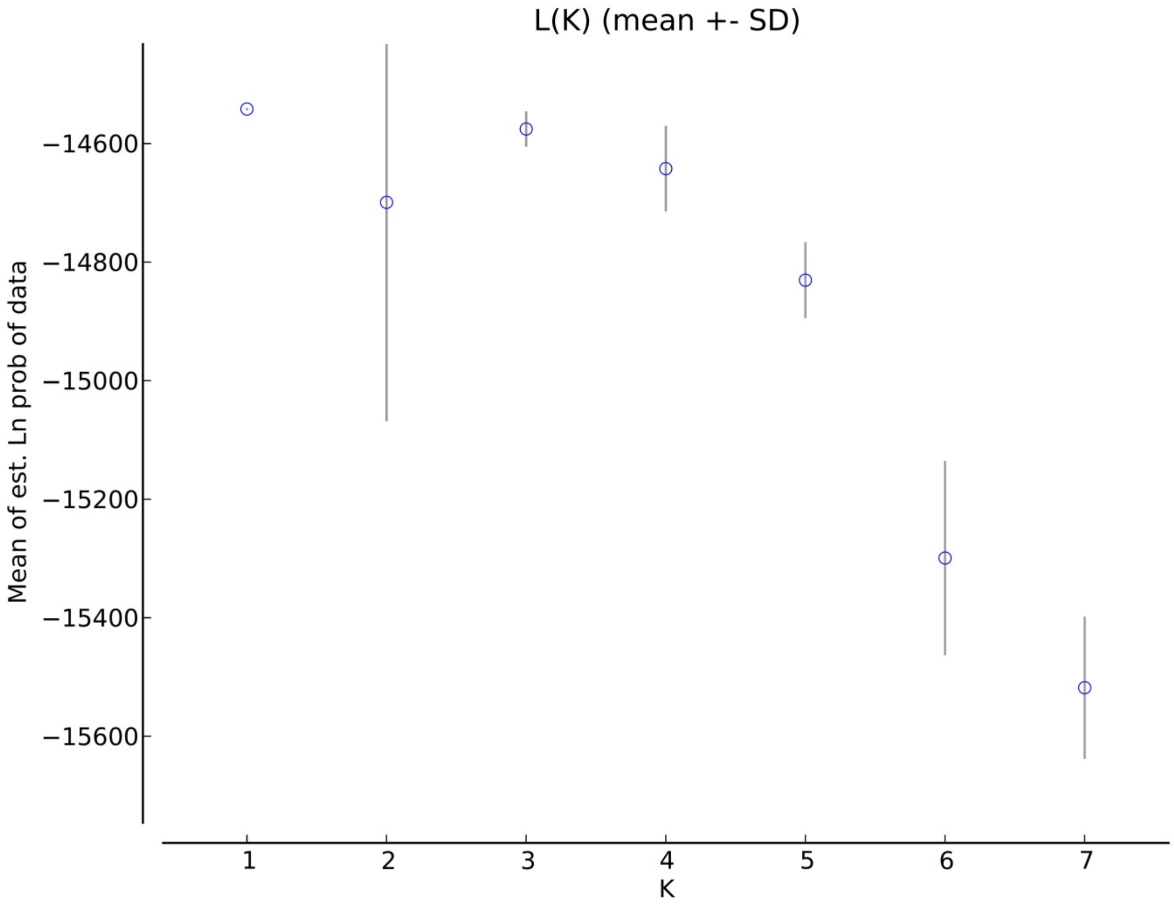

Fig. S2:

LOSITAN outlier detection for the SNPs and Eda marker. F_{ST} for each locus is plotted against expected heterozygosity. Dashed lines denote the upper and lower 95% confidence intervals.

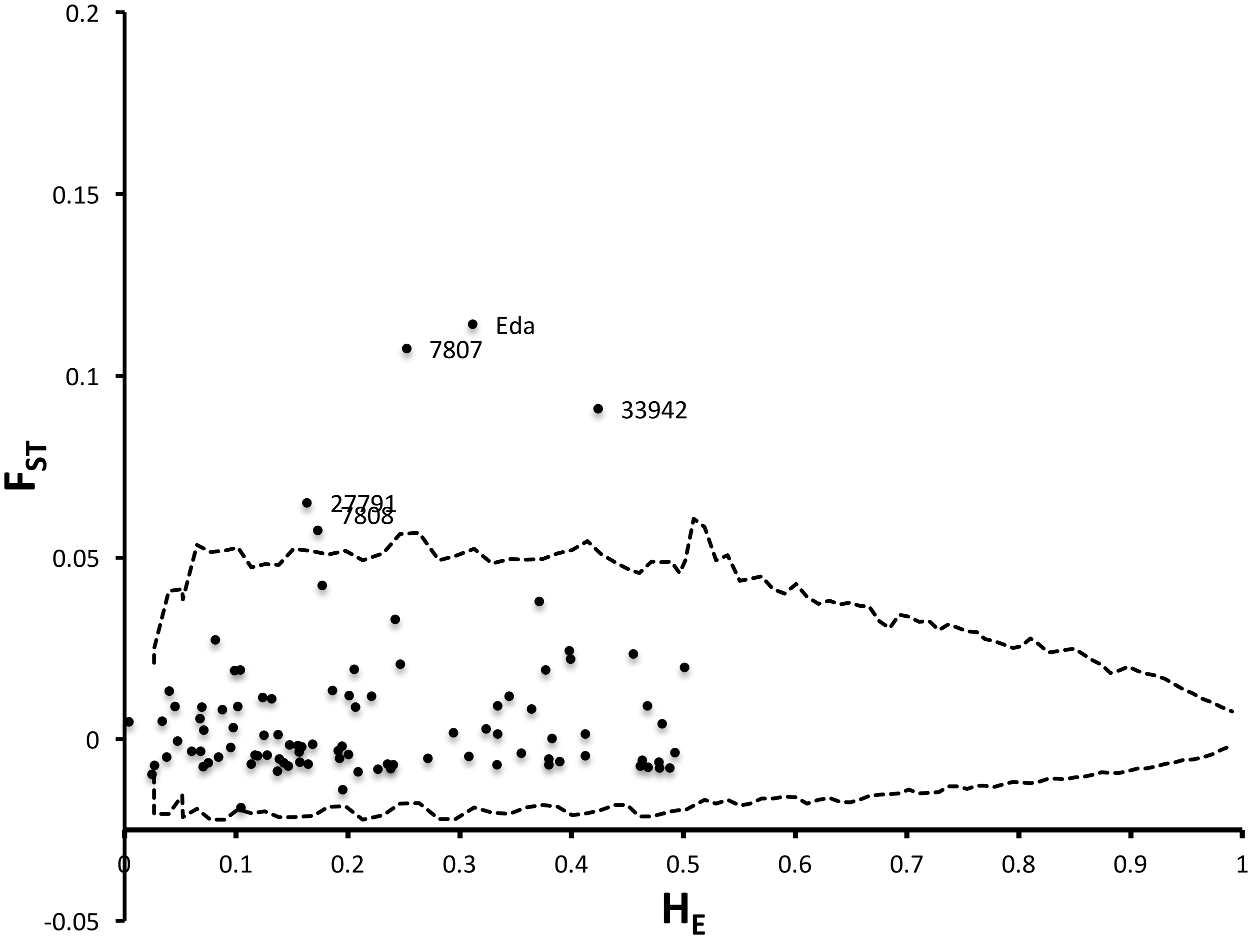

Supplement: Additional file 1: Table S1. — Nonparametric Kruskal-Wallis tests for differences in morphological traits among samples of threespine sticklebacks. Table S2. Nonparametric comparisons for all pairs of samples (Nor, OddM, OddU) using the Steel-Dwass method. Table S3. The principal components for analysis of morphological traits (body shape landmarks), their eigenvalues, the percentage each account for, and 95% bootstrapped confidence (1000 bootstraps) for each. Table S4. Principal component loadings on the three first axes (PC1, PC2, PC3) for each of the 16 morphological landmarks (x and y coordinate). Table S5. Tests for differences in parasite infestation in the trunk among the major groups of samples. Table S6. Tests for differences in parasite infestation (black spot disease) in the trunk among the major groups of samples. Table S7. Fisher’s Exact Test for pairwise comparisons of the three major groups (OddM, Nor, OddU) and their level of parasite infection by black spot disease. Table S8. Cline analysis. Best fit models for SNPs and quantitative traits. Figure S1. Probability of the number of clusters (k) represented by the individual, estimated using STRUCTURE v.2.3.4. Figure S2. Results of outlier tests for the SNPs and Eda marker. (PDF 569 kb) [file 12862_2017_982_MOESM1_ESM.pdf]
